# The Overlap Syndrome: A Combination of Chronic Obstructive Pulmonary Disease and Obstructive Sleep Apnea

**DOI:** 10.7759/cureus.52349

**Published:** 2024-01-16

**Authors:** Mohammad A Alhajery

**Affiliations:** 1 Department of Internal Medicine, College of Medicine, Imam Mohammad Ibn Saud Islamic University (IMSIU), Riyadh, SAU

**Keywords:** chronic obstructive pulmonary disease, obstructive sleep apnea, management, diagnosis, overlap syndrome, obstructive sleep apnea syndrome, copd: chronic obstructive pulmonary disease

## Abstract

Chronic obstructive pulmonary disease (COPD) is a severe lung disease that results in persistent and progressively worsening airflow obstruction due to abnormalities in the airway and alveoli. Obstructive sleep apnea (OSA) is a critical condition characterized by obstructive apneas, hypopneas, and respiratory effort-related arousals. These events occur due to the repetitive collapse of the upper airway during sleep, and it is essential to address this condition. These two conditions, when co-occur, are known as overlap syndrome (OS), which is associated with a higher likelihood of morbidity and mortality compared to either condition alone. Effective management of overlap syndrome is critical to maintain normal oxygen levels during sleep and reduce the incidence of hypoxemia and hypoventilation while improving sleep quality. Positive pressure ventilation is a standard technique used to effectively lower hospitalizations, emergency room visits, moderate and severe exacerbations, and related healthcare expenses in patients diagnosed with COPD and OSA. Despite the lack of literature on overlap syndrome, it is imperative to understand that this condition requires prompt and effective management to prevent further complications. Therefore, this review provides a detailed discussion highlighting the importance of proactive measures to manage overlap syndrome.

## Introduction and background

Chronic obstructive pulmonary disease (COPD) is a lung condition that causes persistent respiratory symptoms such as coughing, expectoration, dyspnea, and exacerbations. COPD affects the airways, causing abnormalities such as bronchitis and bronchiolitis, as well as the alveoli, which can lead to persistent and often progressive airflow obstruction [1,2]. Chronic bronchitis (CB) is the progressive inflammation that occurs in small airways, resulting in obstruction and subsequent airflow limitation. It is important to note that airflow limitations develop slowly, often due to ongoing exposure to hazardous particles or gases that trigger an inflammatory reaction in the body. This reaction is primarily induced by smoking cigarettes, which is also the primary cause of the condition [3]. The diagnosis of CB is clinically confirmed when a person presents a persistent cough for a minimum of three months annually for two or more consecutive years. The term "emphysema" describes the atypical dilatation and destruction of the respiratory bronchiole, alveolar sacs, alveolar ducts, and alveoli, which are the structures distal to the terminal bronchioles. The majority of COPD patients exhibit variable degrees of both CB and emphysema, while some patients may present with only one of these conditions [4]. While primarily impacting the respiratory system, coronary pulmonary disease also has many notable systemic implications, such as impaired skeletal muscle function, nutritional imbalances and subsequent weight loss, and cardiovascular and neurological complications [5,6].

Obstructive sleep apnea (OSA) is a disorder distinguished by obstructive apneas, hypopneas, and arousals related to respiratory efforts, all arising from the repetitive collapse of the upper airway throughout sleeping time. Clinically, most OSA patients present with daytime sleepiness or respiratory disturbances reported by their bed partner during sleep, including gasping, loud snoring, snorting, interruptions in breathing, or choking. Nocturnal restlessness, nocturia, nonrestorative sleep, sleep maintenance insomnia, and morning migraines are less common symptoms [7].

After clinical evaluation, the diagnosis and assessment of OSA severity rely on the frequency of respiratory events during sleep, such as hypopneas, apneas, and respiratory effort-related arousals (RERAs). This assessment generally uses polysomnography or a home sleep apnea test (HSAT) [8].

OSA has a prevalence of approximately 20-25% in the US population [9]. Neuropsychiatric dysfunction, cerebrovascular and chronic vascular diseases, non-alcoholic fatty liver disease, pulmonary hypertension, obesity, metabolic syndrome, and gastroesophageal reflux are among the adverse outcomes that are associated with this condition [10,11]. OSA also tends to increase perioperative complications [12].

The condition in which COPD and OSA coincide is known as overlap syndrome (OS). David Flenley was the one who introduced the term in 1985, with the firm belief that this syndrome had clear and distinct clinical characteristics that set it apart from both COPD and OSA when experienced alone [13]. Flenley also emphasized that the prognosis, progression, and necessity of therapy for this syndrome were equally distinctive [14]. The primary determinants of OSA in individuals with COPD are smoking and obesity [15].

The prevalence of COPD combined with OSA ranges from 0.5% in moderate COPD patients to 39% in US veterans [16]. Prevalence was up to 65% in moderate/severe COPD patients [17]. About 10-30% of COPD patients have mild to severe OSA [18].

In 2020, Adler et al. evaluated differences between patients with OS and those with moderate-to-severe OSA alone. They found that out of 16,466 patients, 14,368 (87%) had moderate-to-severe OSA alone, and 2098 (13%) had OS. A lower percentage of OS patients reported morning headaches, daytime tiredness, and snoring than OSA alone [19].

## Review

Pathophysiology and pathogenesis

While there is currently no established pathological connection between COPD and OSA, the coexistence of these disorders results in heightened nocturnal desaturation due to the combined and mutually reinforcing impact of both conditions. Extensive research has revealed that one of the main reasons behind sleep disturbances in individuals suffering from COPD is nocturnal oxygen desaturation (NOD). This phenomenon arises due to the flattening of the diaphragm caused by persistent obstruction in the airways, which leads to reduced respiratory movement and increased dead space ventilation [14,17-19].

Additionally, obesity, seen in 25-42% of COPD patients, increases the risk of upper airway narrowing, ventilatory dysfunction, and gas exchange abnormalities during sleep [20]. Recent studies have established that the average decline in ventilatory drive during sleep can lead to alveolar hypoventilation, reduced end-expiratory volume, and poor ventilation-perfusion matching in COPD patients, particularly during rapid eye movement (REM) sleep. The medical community widely accepts these findings and forms a critical aspect of managing sleep-related issues in COPD patients [21,22]. Furthermore, sleep also decreases upper airway dilator muscle tone, narrowing the airway and limiting inspiratory airflow, which can further compromise ventilation and increase the partial pressure of carbon dioxide (PaCO2) [23].

Narrowing of the upper airway in OSA exacerbates preexisting hypoxia and ventilatory dysfunction in patients with severe COPD, leading to further oxygen desaturations. Increasing lung volumes due to air trapping was associated with improvements in upper airway collapsibility, and this explains why COPD patients exhibiting symptoms of CB have a higher risk of OSA than those presenting with emphysema [24].

Adverse outcomes

The most significant sleep abnormality observed in COPD and OSA patients is nocturnal hypoxemia (NH). Regardless of OSA, NH in COPD patients is associated with increased mortality, as seen in the post hoc analysis of the Nocturnal Oxygen Treatment Trial [25]. OS patients experience higher oxygen desaturation episodes and sleep time with NH and hypercapnia than OSA patients without COPD [26]. Combined OSA/COPD patients exhibit more severe hypoxemia and cardiac arrhythmias during apneic episodes [27]. The identification of significant and "more profound" NH as an apparent negative prognostic factor for chronic vascular disease and mortality in COPD patients with OS is a well-established fact. Numerous studies have confirmed this association, leaving no doubt about the importance of managing hypoxemia in such patients to improve their overall prognosis [28,29].

Kendzerska and colleagues discovered that the simultaneous presence of NH and COPD in individuals, along with suspected OSA, was associated with an elevated risk of CVD events and mortality. However, this synergistic impact was only observed in female patients [30]. Tang and colleagues have conclusively established a significant correlation between hypoxemia, hypercapnia, and reduced lung function in patients with OS and a higher prevalence of cardiovascular disease events [31]. Moreover, patients with OS are at greater risk of severe COPD exacerbation, leading to hospitalization or death [32].

COPD is associated with an increased risk of pulmonary hypertension (PH) compared with OSA with no coexisting lung disease, which is typically associated with mild PH [33]. OSA and COPD combination increases right ventricular remodeling compared to COPD alone, which explains why OS patients are more likely to develop and have more severe PH than patients with just OSA or COPD alone [34,35].

In a study published in 2018, Economou and colleagues compared OS and OSA patients using several different questionnaires, both before and after treatment with continuous positive airway pressure (CPAP). They found that OS patients had more fatigue than OSA patients, although anxiety, depression, and sleepiness were similar in both groups [36,37].

Diagnosis of overlap syndrome

OSA symptoms and signs in COPD patients are similar to those without COPD, including snoring, sleep maintenance insomnia, awakening with a sensation of gasping or choking, morning headaches, daytime sleepiness or fatigue, and poor concentration or memory impairment. Additional features include a large neck circumference, shallow or "crowded airway," and obesity. The presence of conditions like PH, polycythemia, and daytime hypercapnia should raise the clinical suspicion of OSA in COPD. It is crucial to conduct OSA screening for individuals with chronic stable hypercapnic COPD before initiating long-term non-invasive ventilation (NIV) [38].

Screening Questionnaires

COPD patients rarely benefit from screening questionnaires like the Sleep Apnea Clinical Score, Berlin Questionnaire, and Epworth Sleepiness Scale [38,39]. It is important to note that these methods cannot be solely relied upon to validate COPD patients [39].

Overnight oximetry can serve as a convenient screening tool for identifying nocturnal hypoxia occurring during sleep. The presence of a cyclical (sawtooth) pattern observed can potentially serve as an indicator of OSA in individuals with COPD. However, it is crucial to validate this observation with polysomnography studies [40].

Polysomnography

Polysomnography is unequivocally the most reliable and accurate approach to identifying sleep disorders in people with COPD. Indications for performing a study on sleep in COPD patients are not formally regulated. It is imperative to conduct a full-night polysomnography test for patients with COPD, particularly those with severe COPD and baseline hypercapnia, due to several compelling reasons. The whole night study would increase the chance of detecting different sleep-related disorders in COPD patients, such as OSA, central sleep apnea (CSA), and nocturnal hypoventilation [41]. Additionally, COPD patients, especially those with severe disease, are more susceptible to decompensation and thus have higher hypoventilation and hypoxemia in REM sleep due to muscle atonia/hypotonia. REM sleep frequently dominates the night's second half [42].

In-lab attended polysomnography with positive airway pressure (PAP) titration is the gold standard for diagnosing and treating OS. It is preferable to record PaCO2 non-invasively to detect hypoventilation events and direct PAP titration. For non-invasive PaCO2 monitoring during sleep, transcutaneous CO2 monitoring is the preferable method. This alternative method for monitoring CO2 levels during PAP therapy is more convenient than end-tidal CO2 monitors, which can cause interference and disrupt PAP titration using a mask interface [43]. It is possible that portable and home OSA testing (also known as HSAT) have sufficient diagnostic sensitivity to diagnose OSA in COPD patients. Patients who suffer from severe chronic respiratory diseases should refrain from using HSAT according to the recommendations contained in the most recent guidelines [44]. One study found a high rate of recording failures with HSAT and variable correlation with in-laboratory polysomnography in COPD patients with Global Initiative for Chronic Obstructive Lung Disease (GOLD) stage 2 or 3 and symptoms of OSA [44]. The inability of HSAT to distinguish hypoventilation as the underlying cause of hypoxemia in these patients may result in the administration of inappropriate oxygen therapy or the prescription of an incorrect PAP.

Management of overlap syndrome

Research studies have established a strong correlation between the presence of OS and a higher risk of morbidity and mortality compared to each disease in isolation. Moreover, there is compelling evidence to suggest that implementing therapeutic interventions for OSA in individuals with COPD can significantly reduce cardiovascular mortality and improve overall survival rates among this patient population [45]. The objectives of managing OS include the preservation of normal oxygen levels during sleep and decreasing occurrences of hypoventilation and hypoxemia, along with increasing sleep quality.

Effect of Positive Airway Pressure

PAP is the mainstay of therapy for OSA and OS. CPAP ventilation is generally the first line for treatment of OSA in OS, given the patient has no daytime hypercapnia and features suggestive of nocturnal hypoventilation. The initiation of CPAP in such patients can be through either in-lab titration or by using auto-titrating CPAP with adjustment of pressure and settings as needed. A study published in 2022 by Sterling and colleagues showed that PAP therapy in OS is associated with reduced all-cause hospitalizations and emergency room visits, severe acute exacerbations, and healthcare costs [46].

CPAP therapy significantly improves the partial pressure of oxygen, PaCO2, forced expiratory volume, and mean pulmonary artery pressure [47]. According to the findings of Marin and his colleagues, treatment of OSA can reduce the risk of mortality in individuals with OS. Additionally, it helps lower COPD exacerbations in OS patients. In patients diagnosed with OS and experiencing daytime hypoxia, the utilization of CPAP in conjunction with long-term oxygen therapy (LTOT) resulted in a greater five-year survival rate compared to the use of oxygen therapy alone (71% versus 26%) with higher mortality in patients with OS not treated with CPAP [32].

Research has established a positive correlation between the application of CPAP ventilation therapy and improved survival rates in overlapping hospitalized patients. Voulgaris et al., in a 2023 study, assessed the effect of CPAP compliance on acute exacerbations related to COPD, symptoms, and pulmonary function in OS patients and determined that patients with OS who had substantial compliance with CPAP treatment experienced a reduced incidence of acute exacerbations associated with COPD. Additionally, these patients exhibited enhanced lung function and decreased COPD-related symptoms compared to individuals with poor CPAP compliance [48].

Patients with advanced COPD who exhibit daytime hypercapnia and suggestive signs of nocturnal hypoventilation should undergo an in-lab titration study to initiate PAP therapy unless they are already under NIV therapy. Long-term NIV may enhance physiological indicators like lung function and gas exchange, clinical symptoms like functional capacity, dyspnea, quality of life, sleep quality, and patient-centered outcomes like hospital readmission and survival [38].

Non-invasive Ventilation

Studies have shown that NIV can effectively improve both NH and the quality of sleep. These results suggest that NIV can be a valuable treatment option for individuals experiencing these conditions [49]. These patients benefit from increased strength and endurance of their respiratory muscles due to this treatment. The use of NIV for extended periods in patients with COPD helps in improving lung compliance, preventing atelectasis, and reducing labor that is required to breathe. Despite the extensive research on NIV for treating COPD, there is a significant lack of studies on its effectiveness in managing OS or OSA.

Effect of Supplemental Oxygen Therapy

COPD patients with severe daytime hypoxemia (partial pressure of oxygen ≤ 55 mmHg (7.32 kPa) or oxygen saturation ≤ 88%) can benefit from nocturnal oxygen therapy and qualify for LTOT. The Nocturnal Oxygen Therapy Trial (NOTT) and Medical Research Council (MRC) trials suggest that LTOT improves survival in such individuals [50-52]. Most COPD patients with sleep-related desaturation and no significant daytime hypoxemia do not benefit from nocturnal oxygen.

Nocturnal oxygen may reduce NOD episodes but not sleep quality or nighttime arousals [53]. In a trial where 20 men with OS received four liters of supplementary oxygen each, an improvement in NOD despite an increase in the frequency of obstructive episodes associated with hypercapnia and a reduction in pH was observed. As a result, the use of supplemental oxygen therapy as a definitive therapeutic option for OS is not suggested [54].

Effect of Pharmacotherapy

COPD treatment can lead to a decrease in NOD and the requirement of oxygen supplementation therapy. Martin et al. [55] studied patients with moderate to severe COPD. They found that inhaled ipratropium, administered four times daily, significantly improved their NOD, sleep quality, and total time spent in REM sleep. The study also revealed that long-acting beta-agonist medication and oral corticosteroid therapy for COPD patients had similar beneficial effects. On the other hand, less is known about whether or not treating COPD in a patient with OS can improve OSA symptoms [56].

How lifestyle modifications help in managing COPD

Studies have shown that structured exercise programs and pulmonary rehabilitation can provide considerable benefits to individuals affected by both OSA and COPD. These interventions can help improve lung function, reduce symptoms, and enhance the overall quality of life in patients with these conditions. The primary objective of structured exercise programs is to reduce skeletal muscle wastage in COPD individuals. Similarly, implementing a structured exercise program in individuals with OSA has demonstrated enhancements in several key areas, including the apnea-hypopnea index (AHI), daytime sleepiness, and overall sleep quality. It is crucial to recognize that depending solely on a predetermined exercise program is inadequate to attain optimal outcomes in disease management. Thus, it is highly recommended to complement it with suitable therapeutic interventions to guarantee the most favorable results. Patients diagnosed with COPD have reported experiencing enhancements in several aspects of their well-being, including the quality of life, mood index, dyspnea ratings, and a decrease in hospital visits, as a result of engaging in pulmonary rehabilitation [57]. A clinical trial evaluated the impact of pulmonary rehabilitation in a cohort of 64 individuals diagnosed with COPD. Based on the Pittsburgh Sleep Quality Index, a considerable percentage of the participants, precisely 58%, reported experiencing poor sleep quality initially. However, after the eight-week intervention period, this percentage decreased significantly to 19%. Obese patients must prioritize weight loss and quitting smoking to substantially reduce the morbidity and fatality rates associated with the disease [58].

Future trends

Researching to define the prevalence of OSA and COPD in the OS population is of paramount relevance. This step will facilitate the development of clinical studies aiming at phenotype-directed therapeutic approaches and their implementations in the context of PAP therapy. Further investigation is required to assess the significance of in-lab polysomnography in the titration of NIV for patients with chronic hypercapnic respiratory failure due to COPD [38].

In the most recent evidence, the administration of nocturnal oxygen to COPD patients who had NH did not lower mortality or the progression to LTOT. On the other hand, it is not possible to rule out the possibility that these patients have sleep-related hypoventilation. Therefore, the relevance of diagnosis and treatment of other sleep-related breathing disorders in COPD patients who have borderline and nocturnal hypoxemia is another exciting research field that might be explored [59].

OS requires research into more recent NIV techniques. The auto-trilevel PAP is an innovative, non-invasive modality developed exclusively for OS patients. This mode uses a lower expiratory positive airway pressure (EPAP) at the beginning of expiration to mitigate the effects of auto-positive end-expiratory pressure without generating dynamic hyperinflation. It utilizes a greater EPAP toward the end of expiration, during which upper airway collapsibility is most likely to occur [60]. Preliminary research has indicated that it may be more productive than bi-level positive airway pressure in decreasing AHI, NH, time spent sleeping ineffectively, and daytime sleepiness. In COPD patients with persistent hypoventilation, volume-assured pressure support (VAPS) combined with fixed or auto-EPAP is helpful in some limited studies. It can ensure suitable minute ventilation even though the patient's ventilatory drive and effort can change depending on the stage of sleep they are in and their posture [61].

## Conclusions

Overlap syndrome, a combination of OSA and COPD, is associated with higher morbidity and mortality compared to either condition alone. It is crucial to screen and test for OSA in patients with COPD and, conversely, assess COPD in patients with it. The management of overlap syndrome requires the treatment of both clinical conditions simultaneously, with PAP being a crucial component in decreasing morbidity and mortality. Additionally, optimizing COPD treatment and implementing interventions such as quitting smoking for smokers and weight loss for obese patients are essential. However, there is a need for more research in this area, mainly focusing on phenotype-directed diagnostic and management approaches.
