# Correction: The Overlap Syndrome: A Combination of Chronic Obstructive Pulmonary Disease and Obstructive Sleep Apnea

**DOI:** 10.7759/cureus.c155

**Published:** 2024-01-24

**Authors:** Mohammad A Alhajery

**Affiliations:** 1 Department of Internal Medicine, College of Medicine, Imam Mohammad Ibn Saud Islamic University (IMSIU), Riyadh, SAU

This article has been corrected to accurately reflect the affiliation of the author. The affiliation has been changed from Internal Medicine/Pulmonary Medicine and Sleep Medicine, Imam Mohammad Ibn Saud Islamic University, Riyadh, SAU to Department of Internal Medicine, College of Medicine, Imam Mohammad Ibn Saud Islamic University (IMSIU), Riyadh, SAU. The author deeply regrets that this error was not identified and addressed prior to publication.

 

